# The climatic risk of Amazonian protected areas is driven by climate velocity until 2050

**DOI:** 10.1371/journal.pone.0286457

**Published:** 2023-06-22

**Authors:** Calil Torres-Amaral, Luciano Jorge Serejo dos Anjos, Ima Célia Guimarães Vieira, Everaldo Barreiros de Souza

**Affiliations:** 1 Postgraduate Program in Environmental Science—PPGCA, Institute of Geosciences, Meteorology Faculty, Federal University of Pará—UFPA, Belém, Pará, Brazil; 2 Postgraduate Program in Ecology and Conservation, State University of Mato Grosso, Nova Xavantina, Mato Grosso, Brazil; 3 Campus Parauapebas, Federal Rural University of the Amazon, Parauapebas, Pará, Brazil; 4 Botany Department, Museu Paraense Emilio Goeldi, Belém, Pará, Brazil; Universidad Cooperativa de Colombia, COLOMBIA

## Abstract

Changes in species distribution in response to climate change might challenge the territorial boundaries of protected areas. Amazonia is one of the global regions most at risk of developing long distances between current and future analogous climates and the emergence of climate conditions without analogs in the past. As a result, species present within the network of Protected Areas (PAs) of Amazonia may be threatened throughout the 21st century. In this study, we investigated climate velocity based on future and past climate-analogs using forward and backward directions in the network of PAs of Amazonia, in order to assess the climatic risk of these areas to climate change and verify their effectiveness in maintaining the current climate conditions. Using current (1970–2000) and future (2041–2060) average annual air temperature and precipitation data with a resolution of 10 km, climate velocities across the entire Amazon biome and average climate velocities of PAs and Indigenous Lands (ILs) were evaluated. The results show that the effects of backward velocity will be greater than that of forward velocity in the Amazon biome. However, the PA network will be less exposed to backward velocity impacts than unprotected areas (UAs)–emphasizing the importance of these areas as a conservation tool. In contrast, for the forward velocity impacts, the PA network will be slightly more exposed than UAs–indicating that the current spatial arrangement of the PA network is still not the most suitable to minimize impacts of a possible climate redistribution. In addition, a large extent of no-analog climates for backward velocities was found in central Amazonia, indicating that high temperatures and changes in precipitation patterns in this region will surpass the historical variability of the entire biome, making it a potentially isolated and unsuitable climatic envelope for species in the future. Most of the no-analog climates are in PAs, however the climate risks in ILs should also be highlighted since they presented higher climate velocities than PAs in both metrics. Our projections contrast with the median latitudinal migration rate of 2 km/year observed in most ecosystems and taxonomic groups studied so far and suggest the need for median migration rates of 7.6 km/year. Thus, despite the important role of PAs and ILs as conservation tools, they are not immune to the effects of climate change and new management strategies, specific to each area and that allow adaptation to global changes, will be necessary.

## Introduction

Climate change is predicted to become the most serious threat to biodiversity in the 21st century [1]. Among the numerous and multifaceted dimensions of climate change, there is the redistribution of climatic conditions over large spatial extensions [2, 3]. Given the spatial scale of this phenomenon, it’s impact on biodiversity is commonly related to the macroecological effect of reducing (or expanding in some cases) the geographic distribution of a species [4]. This impact directly affects the static way in which biodiversity conservation strategies are currently developed. Despite the demarcation of protected areas (PAs) and indigenous lands (ILs) being considered the most efficient and economical line of defense in the global effort to protect biodiversity [5–7], and although its effectiveness in controlling deforestation and other anthropogenic pressures of land use and land cover is recognized [8], even the natural communities existing in these territories are not immune to climate change.

Across the Amazon, an extensive network of 6983 ILs and 1170 PAs expands over 4.5 million km^2^ and occupies 53.6% of the biome [9]. The carbon stock of the Amazon’s PAs and ILs alone is enough to destabilize or contribute to the stabilization of the Earth’s atmosphere [10]. However, whilst PAs are subject to chronic disturbances such as deforestation and forest degradation [11], evidence suggests that climate change may also limit their efficacy [12]. Ensuring the continued relevance and effectiveness of the PA network across Amazonia is therefore a critical challenge for biodiversity conservation in face of climate change [13, 14].

The spatial redistribution of biodiversity is considered one of the main ecological responses to climate change [15]. This phenomenon is likely to be particularly pronounced across the tropics where species have narrow climatic niches, making them less able to acclimatize or adapt [16], probably due to evolution under stable climatic regimes [17]. Evidence at regional [18] and continental [19] scales suggests that many tropical plant species are already shifting their geographic distributions due to warming from lowlands to Andean slopes.

Among the metrics for assessing the climatic risk of biota to climate change [2], climate velocity stands out as a metric that describes the spatial change of climate variables over time, resulting in a rate of change (km/year), which from a biological perspective describes the speed that an organism needs to migrate to maintain constant climatic conditions [20]. This climatic risk assessment metric has been increasingly used in conservation planning [21], and since it uses only climate data, it is becoming indispensable for megadiverse ecosystems with absence of specific information, such as the Amazonia.

Analog-based climate velocities represent the physical distance between a location and the location where a similar climate will be found in the future. This concept can be employed in two temporal directions: forward velocity–which reflects the distance from a grid cell in a current climate to the nearest cell with similar projected future climate; and backward velocity–which reflects the distance from projected future climate cells back to the closest cell with a similar current climate [20]. The ability to calculate two different metrics allows the extraction of two ecological implications from the same cell. Forward velocity reflects the shortest distance that an organism in the current landscape must migrate to track a consistent climate condition. A high forward velocity indicates a potential risk for climatically-adapted species, as they may be unable to migrate at the necessary speed (km/year). Moreover, a climate that lacks a future analog is classified as a "disappearing climate" [3, 12]. Species inhabiting cells with high forward velocity or disappearing climates are ultimately under threat of extinction. Alternatively, backward velocity reflects the shortest distance that an organism in the current landscape would have to migrate to colonize a particular newly suitable habitat given the projected future climate. A high backward velocity indicates a potential risk for sites on holding species adapted to its future climate. Accordingly, a future climate that lacks a current climate analog is classified as a "novel climate". Therefore, cells with high backward velocity or novel climates are ultimately under threat of becoming unsuitable habitats, with consequent effects on ecosystem function and services. Whilst forward velocity measures the distance from a single origin to multiple future destinations, backward velocity implies the distance between multiple origin points to a single future destination. Consequently, the use of these two metrics in planning will result in a contrasting focus on species/populations (forward velocity) or sites (backward velocity) [20, 21].

The ecological interpretation of the effects of climate velocity is often associated with the movement of species, which relies on the assumption that species will not be able to acclimate or adapt to shifting climate and thus need to move to track suitable climates. Observed evidence from long-term forest dynamics in Costa Rica, Panama and Malaysia indicated that trees have declining growth rates in response to regional increases on temperatures [22, 23]. Further, evidence from a long-term drought experiment in the Amazon indicated no change in the overall thermal sensitivity of net photosynthesis or foliar respiration rates in response to reduced precipitation [24]. Both studies indicate that individuals may be unable to acclimate physiological processes to changing climate. Similarly, long‐term inventory plots have indicated that long generation times of tree communities are lagging behind increases in moisture stress [25]. By contrast, a long-term collection of morphometric data on a nonmigratory understory bird community within Amazonia suggested that other components of biodiversity are potentially already responding to climate change through morphological shifts [26]. In this work, the potential ecological implications of the climate change velocity metric will be discussed assuming the premise that most species will not be able to acclimatize and, therefore, will respond to climate change through spatial changes.

To understand the underlying causes that determine the climate risk of different areas, climatic velocities provide two valuable pieces of information. For example, for areas that have analogous climates, the magnitude of velocity is determined by the distance between analogous climates. In this context, climate velocities depend, to an extent, on the geography of the study area, which was not investigated in this study. Alternatively, for areas that have no-analog climates, climate risk is determined by the magnitude of the climatic variables used, as their values ​​escape the historical variability of the study region. As the multivariate definition of climate analog was used, there is no single temperature or precipitation threshold that determines the presence of no-analog climates. Rather, the definition of no-analog is a result of the lack of equivalent precipitation and temperature values ​​within the historical variability of all cells within the study area.

The Amazon has been ranked amongst the global regions most at risk of developing new climatic conditions [3], and developing the greatest distances between current and future (2100) equivalent temperatures [27]. Previous velocity studies carried out at macroclimatic scale and considering only temperature, have overestimated velocities in regions close to the equator, driven by the dominance of flat areas [28]. The only visualization [20] of the spatial distribution of analog-based climate velocity for the Amazon to date is at macroclimatic scale (50x50 km). The application of finer scales such as meso (1-10km), topo (10m-1km) or microclimatic (<50m) is therefore essential to identify refuges and other areas where conservation may facilitate the persistence of biodiversity under climate change, even within potentially unsuitable regions [29]. Using the 5km x 5km scale, other studies [12, 30] have sought climatic analogs using forward velocity, first across the entire Brazilian Amazon incorporating deforestation scenarios, and then focusing on the edge of the PA network. However, the variation in magnitude of climate risks across PAs were not calculated, nor were the backward velocity calculated. More recently, forward velocity was associated with ecological niche models, but as one of several measures to identify priority areas for mammals [31].

In view of the high rates of exposure to climate change previously projected across the Amazon, and given its global importance as a hotspot for biodiversity [32], we ask: 1) What is the climatic risk to the network of PAs and ILs across the Amazon in terms of climate redistribution that will occur until 2050 under the IPCC SSP245 scenario?; 2) Will the highest levels of climate risk be inside or outside of the PAs and ILs?; 3) Will the climate risk levels be higher in ILs or PAs? We work with the hypotheses that: the Amazon biome will be exposed to high rates of forward and backward velocities and will have a large area without climatic analogs; however, significant differences in climate risk between PAs, ILs and UAs, will be found. These differences are expected to infer different ecological implications across Pas, ILs and UAs, and consequently necessitate the implementation of different conservation strategies across the Amazon.

## Material and methods

### Study area

We used the biogeographic boundary outlined by the Amazon Network of Georeferenced Socio-Environmental Information [33] (RAISG, September 2020, http://raisg.socioambiental.org/) (S1 Fig) to define the study region of the Amazon. This region covers approximately 7 million square kilometers and is characterized by a high diversity of ecoregions (53) [34]. The area has predominantly low topographical diversity, with some isolated areas of elevations distributed across eight countries (Bolivia, Brazil, Colombia, Ecuador, Guyana, Peru, Suriname, and Venezuela) and one French territory (French Guiana). To define the network of protected areas (PAs) and Indigenous Lands (ILs), we used spatial data from World Database on Protected Areas (WDPA, September 2020, https://www.protectedplanet.net) [35]. This database includes terrestrial and marine PAs administered by national, regional, or international entities, representing an ecologically based protection system for all countries throughout the world. We considered all terrestrial PAs across the Amazon, totaling 837 PAs covering 3,111,929 km^2^. We excluded PAs defined by international entities such as RAMSAR sites, biosphere reserves, and world heritage sites. Overall, PAs and ILs cover about 44.8% of the study area, of which 42.7% are ILs.

## Climate data

We used temperature and precipitation data from WorldClim, a repository that provides spatially interpolated monthly climate data for global land areas at a high spatial resolution (approximately 1x1 km) [36]. To apply the climate velocity metric and make inferences about the spatial distribution of living organisms [29, 37], we used a 10x10 km resample provided by WorldClim. At this scale, the climate is determined by factors ranging from large-scale continental circulation patterns to the local variability of the terrain, which is fundamental for our analysis. We selected mean annual temperature (Bio1) and annual precipitation (Bio12) as the bioclimatic variables because they represent biologically relevant annual trends. Mean annual temperature is an important indicator variable for a set of other temperature-based metrics [38] and produces projections with relatively lower levels of uncertainty compared to precipitation [39]. Total annual precipitation on the other hand, is crucial for understanding the functioning of the Amazon ecosystem [40]. The current climatic conditions were based on the average values recorded from a reference climatic period of 1970–2000 [36]. To represent future climate data, we used an ensemble of 5 GCMs (S1 Table) selected from the World Climate Research Program Coupled Model Intercomparison Project phase 6 (CMIP6) for the period 2041–2060 under a greenhouse gas concentration scenario SSP245. This scenario predicts an average trajectory within the range of future paths where trends continue their historical patterns without substantial deviations. This condition has the potential to reflect a minimum-gain trajectory of climate governance, where societies rapidly reduce emissions but not enough to limit warming to below 2°C. In practice, some countries may reach and increase in temperature of 3°C by the end of the 21st century–, which is above the objective established in the Paris Agreement of 2015 [41, 42].

### Forward and backward velocities

We used an algorithm based on analogous climates to calculate the forward and backward climatic velocities [43, 44]. The algorithm determined the trajectories with the least climatic dissimilarity between current (annual averages) and future projections, using the least-cost path configuration. To define the specific analogous climate for each cell, we examined the set of values covered by the standard deviation of monthly historical variability of average temperature and total annual precipitation of each cell between 1970 and 2000. We measured distances between similar climates using two climatic variables Bio1 and Bio12 by first measuring the distances for each variable and then averaging them. Thus, for each pixel in the study area we calculated the lowest cost path to a cell with similar climatic conditions. A greater distance indicated a greater hazard for the cell in terms of spatial redistribution of climatic conditions. To calculate the forward velocity, we determined the future analog that corresponded to the present values of the climatic variables. Similarly, we calculated the path with the lowest cost between a cell with a climate projected for the future and another with similar conditions in the present to calculate the backward velocity. Cells for which no correspondence could be found in future projections represented “disappearing climates” or “novel climates” when the new climate fell outside the historical annual variability of any other region of the Amazon [3].

### Climate risk assessment

We calculated forward and backward climate velocities for all pixels within PAs and ILs using baseline (1970–2000) and mid-21st century climate (2041–2060) data. We then classified each protected area based on a joint characterization of forward and backward velocities, dividing the range of velocity values into low, moderate, and high using three equal area quantiles. This resulted in a nine-quadrant bivariate plot (S3 Fig). PAs without analogs were those that lacked analogs in any cell. We compared the median velocity of the entire network of PAs and ILs to the median speed in UAs to evaluate the effectiveness of the spatial arrangement of the PAs and ILs system in reducing climatic velocity. Similarly, we compared how median climatic velocities differed between PA and IL categories. We also performed cell-level analyses to compare value variability across different scales of analysis. To quantify the spatial extent of no-analog cells, we counted the number of no-analog cells in each of the PAs and ILs and compared the extensions of no-analogs between UAs, ILs and PAs. Finally, we compared the geographic distribution of the region identified as no-analogous with the distributions of climate deltas (i.e., the difference between present and future mean temperature (2050–1990) classified as low, medium and high) and the historical annual variability (i.e., 1 standard deviation) of precipitation and temperature (from 1970–2000) across cells (S2 Fig). This allowed us to demonstrate the spatial overlap between areas with higher climate deltas and lower historical climate variability and future no-analog cells.

## Results

**Fig 1:**
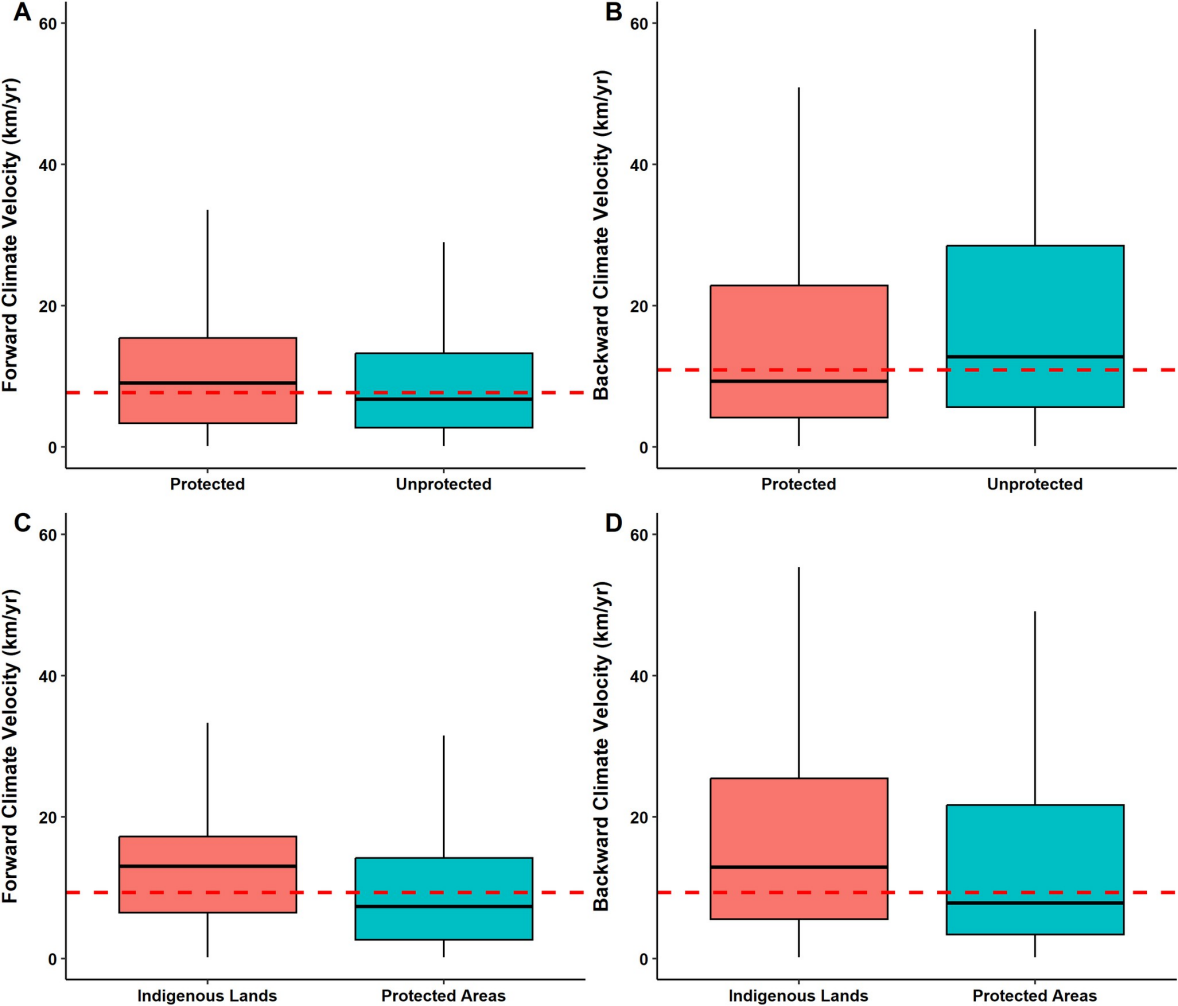
Comparison of forward and backward velocities between protected and unprotected areas (A-B) and Indigenous Lands and Protected Areas (C-D). The dotted red line represents the median value of each of the velocities over the entire Amazon biome.

The comparison between the medians of forward and backward velocities across the Amazon showed a statistically significant difference (W = 1435894876, p-value < 0.001) between the backward velocity (10.9 km /year) and the forward (7.6 km/year). When comparing the velocities inside and outside the PAs and ILs (Fig 1), the medians are significantly different in both forward (W = 715900341, p < 0.001) and backward (W = 300464189, p < 0.001). For forward velocities, the median of protected areas (9 km/year) was higher than that of non-protected areas (6 km/year). On the other hand, for backward velocities, the median of UAs (12.8 km/year) was higher than that of PAs (9 km/year) and ILs (9 km/year).

The comparison of the median climatic velocities between PAs and ILs (Fig 1C, 1D) showed a significant difference between the categories for both forward (W = 166466959, p-value < 0.001) and backward (W = 52366546, p-value < 0.001). At both velocities, the median of ILs was higher than that of PAs. The median forward velocity in PAs is 7 km/year, while in ILs it reaches 13 km/year. The backward velocity is higher for both categories, being 7.8 km/year in PAs and 12.8 km/year in ILs.

**Fig 2:**
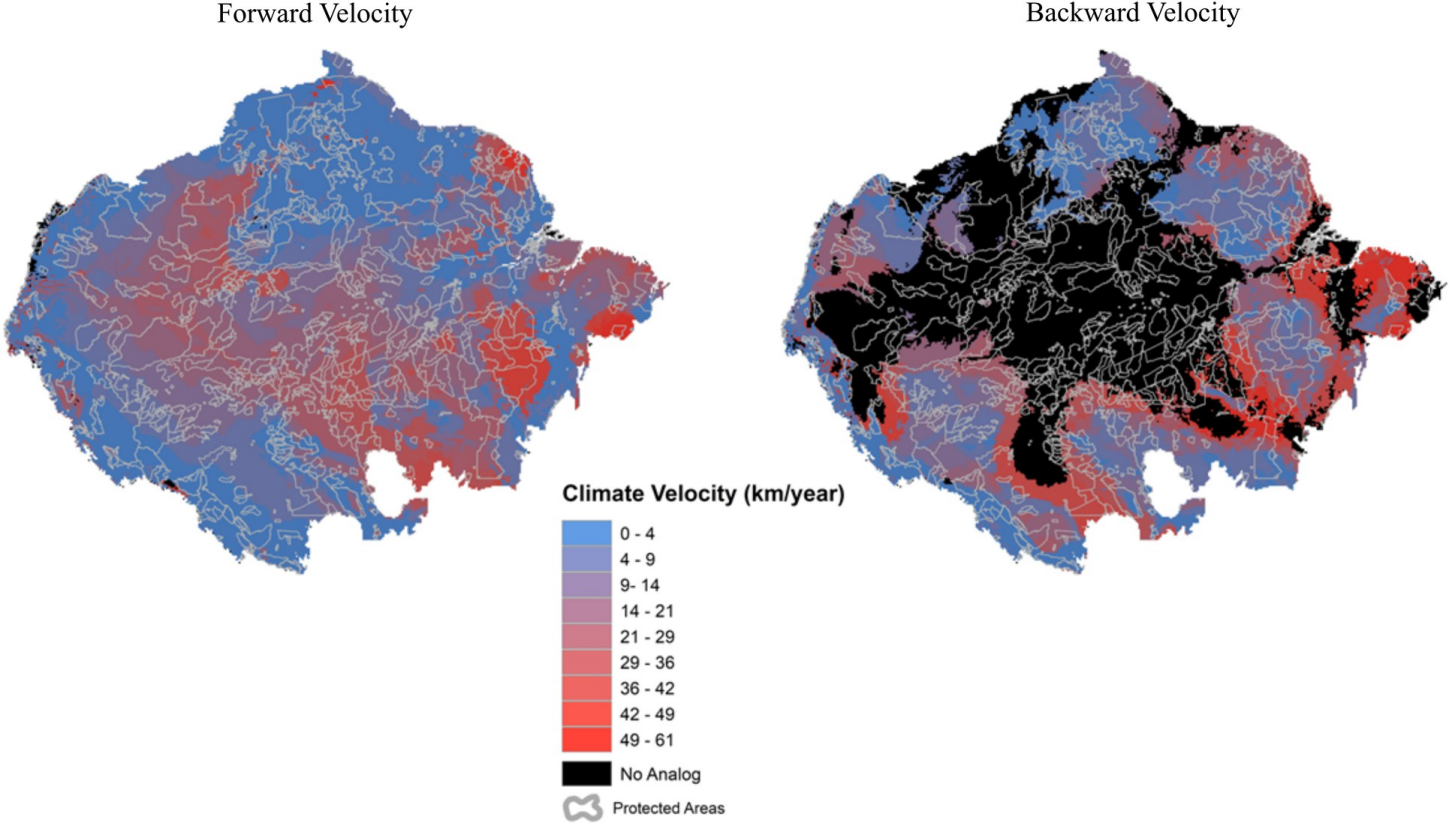
Spatial distribution of forward and backward climate velocities at the cell level. Map source: Amazon biogeographical limit [9]; protected areas and indigenous land [35].

If we consider the presence of no-analog climates (Table 1), our results show that approximately one third (33.5%) of the PAs and ILs do not have a climatic analog in any of the metrics, and 33% of them are due to new climates (no-analog for backward velocities) and 0.5% are for disappearing climates (no-analog for forward velocities). The PAs also varied in relation to the presence of no-analog climates at the cell scale (Fig 2). About 3.2% of the PAs presented at least one cell that did not have a climatic analogs with respect to forward velocity, and almost 45% of the PAs with respect to backward velocity.

**Table 1:** Spatial extent and cover percent of disappearing and novel climates in unprotected areas, protected areas and indigenous lands.

Protection category	Climatic risk	Cover across Amazon (%)	Area (km^2^)
UAs	Disappearing	0.29	20677.66
UAs	Novel	25.44	1774348.06
PAs and ILs	Disappearing	0.15	10702.90
PAs and ILs	Novel	17.70	1235069.07
PAs	Disappearing	0.1522	10617.44
PAs	Novel	11.96	834745.27
ILs	Disappearing	0.0073	515.73
ILs	Novel	6.26	436616.21
Amazon (all area)	-	100	6974270.54

**Fig 3:**
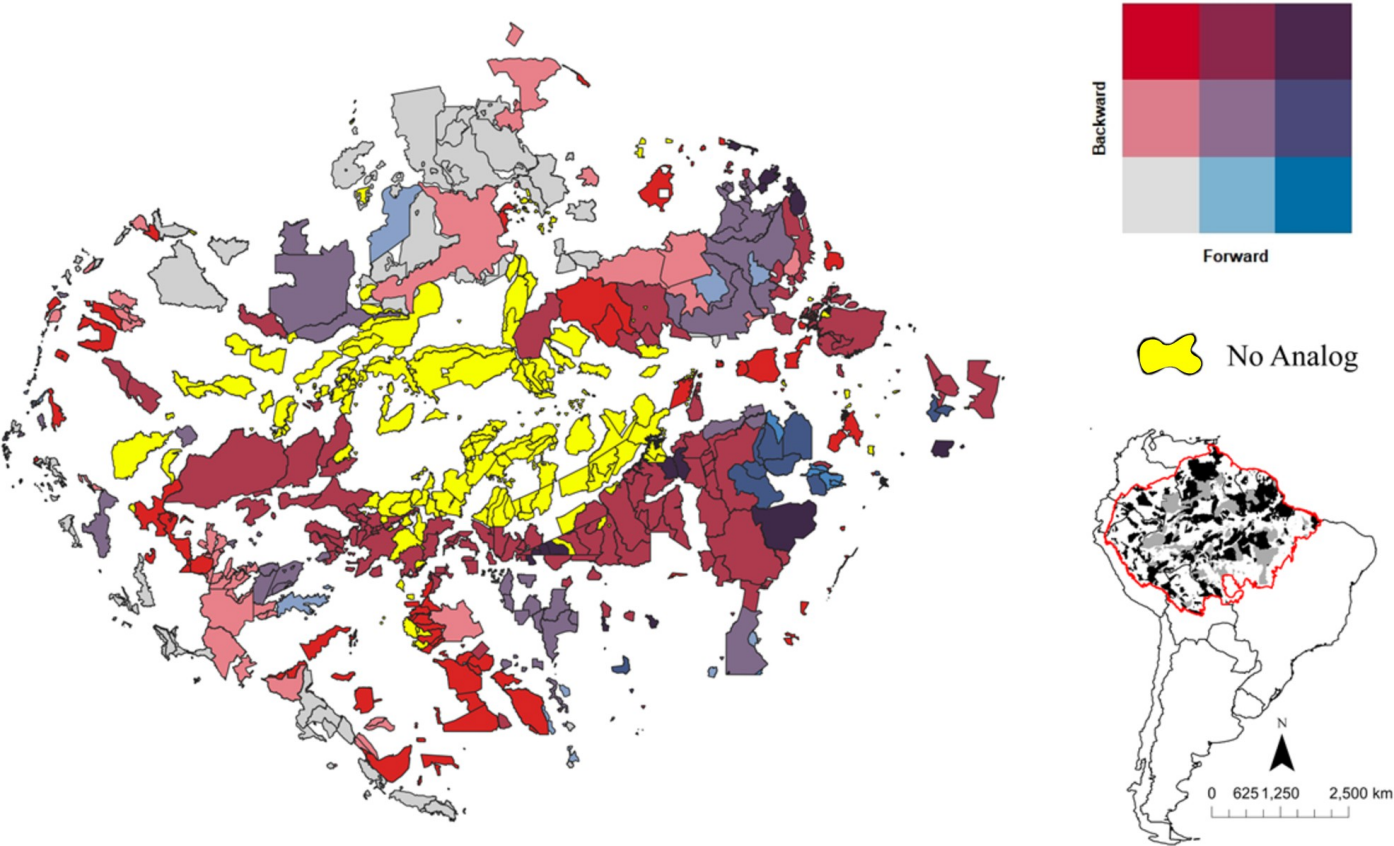
Velocity-based climatic risk assessment of each protected area within the Amazon biome. We assessed the velocity-based climatic risk of each PA within the Amazon based on nine categories derived from the classification of the gradient of values of forward and backward velocities in three equal quantiles of area (in the up-right panel). No-analog PAs and ILs are indicated in black and are not included in the bivariate panel. In (c) we see the biogeographic boundary of the Amazon with PAs in black and ILs in grey. Map source: Amazon biogeographical limit [9]; protected areas and indigenous land [30].

The velocity-based climatic risk assessment shows that the extent of areas with high backward-only velocity (32.4%) is greater than that of forward (5.85%). This trend stands out if we consider the large number of PAs and ILs with low forward velocities (31.3%), in relation to backward (16.3%) (as defined by the quantile with the lowest values within a range of velocities previously divided into three equal areas). In addition, a higher percentage of PAs and ILs experience low forward and backward combined velocities (11.4%) than high combined velocities (3.1%). However, the smaller number of PAs classified with high velocities may underestimate the real threat to which these areas will be subjected to (Fig 3).

## Discussion

When comparing climate velocities across Amazonia we find that the network of PAs and ILs will be less at risk to the impacts of backward velocity compared to UAs, emphasizing the importance of these areas as tools in conservation. In contrast, for forward velocity impacts, the PA network will be slightly more exposed than UAs–indicating that the current spatial arrangement of the PAs and ILs network is still not optimal for minimizing the impacts of climate redistribution. Despite this difference, both PAs, ILs and UAs will be exposed to high rates of climatic velocity, particularly in terms of backward velocity, which will cause greater effects in magnitude considering a median value across the entire Amazon. Previous studies have already classified Amazonia as having a high risk of developing new climatic conditions [3], developing greater distances between current and future (2100) equivalent temperatures [28] and high forward and backward velocities [20]. These projections are much greater than the median latitudinal migration rate of 1.69 km/year observed in most ecosystems and taxonomic groups studied so far [45]. Our results are in line with previously projected climate velocities, however, our projections also reveal the types and magnitudes of particular threats within the Amazon, especially with respect to PAs and ILs.

The potential impacts of both forwards and backwards velocities will be greater on ILs than on PAs. This means that species within ILs, rather than PAS, will be further away from their climate analogs by 2050, and will therefore have to migrate at higher annual velocities, whilst their former climatic envelopes will not become suitable for other colonizing species. Given the correlation between velocities based on analogs and geographic characteristics [37], we suggest that this pattern is the result of the arrangement between the distribution of climatic velocities and the spatial arrangement of the ILs. However, further studies are needed to identify which spatial features determine this pattern.

The same striking pattern of backward velocity risk can be seen in the spatial extent of areas without analogous climates. There is a large distribution of novel climates throughout the central region of the Amazon, making this area unsuitable as a habitat for species that cannot acclimate or adapt, and thus, indicating potential effects on the ecosystem functions and services of floodplain forests [46]. In contrast, few areas have been identified as disappearing climates. The majority of areas with novel and/or disappearing climates are in UAs, however, within the PA and IL network, most novel/disappearing climates are spatially distributed in PAs. Nevertheless, it is important to note that when considering PAs and ILs who’s entire territory will be without a climate analog across Amazonia by 2050, ILs are the majority, reinforcing the pattern of high climate risk found in IL’s.

Based on spatial distribution of forward and backward climate velocities at the cell level, our results show that the backward velocity is gradually distributed from values ​​with lower speeds in some parts of high elevation areas towards the edges of Amazonia, becoming higher in the lowland interiors. In central Amazonia, along the entire floodplain region of the Amazon River, there is a large extension of climatic conditions that are not analogous to the historical variability of the rest of the biome. The forward velocity is higher along a diagonal extending from the central-western region to the southeastern region, but presents some higher velocity hotspots in parts of eastern Amazon.

Under the assumption that species are unable to acclimate/adapt to changing climate [16], these results are a cause for concern, given the significant role of PAs, and especially ILs, on forest conservation, which reduces forest carbon emissions and contributes to climate change mitigation [47]. Many studies have shown that Amazonian PAs and ILs act as buffers for the advance of the deforestation frontier, reducing deforestation [11], degradation [46] and fire occurrence [48]. These results imply that despite the important role of Pas and ILs in containing changes in land use and cover, they are not immune to climate change, and therefore, climate adaptation measures are needed to increase their resilience.

Adaptive management strategies [49] can be applied in view of the impacts on the distribution of species projected by the climate velocity metric. In areas with high forward velocity, climate adaptation strategies are recommended, and in extreme cases, assisted migration [21, 50]. Conservation strategies that prioritize the protection and connectivity of climatically heterogeneous landscapes are also suggested, either through the expansion of existing reserves or the creation of new corridors that facilitate movement in response to climate change [51]. In areas with high backward velocity, intervention through assisted migration is indicated, either in the context of conservation of species with restricted distributions or on a large scale, such as movement of populations of widely distributed species in regular reforestation operations [52]. Actions focused on monitoring invasive alien species that may occupy empty niches may also be necessary [21].

However, comparative analyzes such as those carried out in this work (UAs vs PAs; ITs vs PNAs) are not without limitations. For example, they can mask the real risk that different areas will be subjected to in terms of actual climate velocity values. In our analysis, exposure to moderate and low velocities means a spectrum of 0–13 km/year on forward velocity and 0–21 km/year for backward velocity, as defined by the classes using quantile method. In previous research, PAs in North America were classified as having high climate velocity when they obtained values above 10 km/year [53]. Therefore, here, even the classification of moderate velocities, depending on the dispersion capacity and sensitivity of the species, may indicate high risk. In the same way, whilst the backward velocity for PAs revealed a lower median value than that of UAs, this value was still 12 km/year, which may imply severe threats to some species.

Finally, it should be noted that this work does not take into account the potential influences of changes in land use and cover on species dispersion. However, deforestation and degradation can influence the dispersion of species, both via the formation of barriers that restrict the movement of species between analogous climates [31], as well as potentiating the effects of climate change on local [54], regional [55] and global scales [56]. Also, while any environmental variable can be utilized to calculate climate velocity, temperature is the most commonly used variable in research studies, as it has a significant influence on species distribution at the landscape level. The bioclimatic variables mean annual temperature and total annual precipitation were selected as they represent biologically relevant trends on species distributions. However, it should be also noted that while annual mean values are better suited for predicting changes across the entire range of a species, extreme temperature values are appropriate for predicting a species’ tolerance limits, which represents a promising approach for future studies [21]. The methodological limitations inherent to analyzes based on climate change metrics and the degree of uncertainty of climate projections, both of which are extensively debated should also be kept in mind [57, 58].

## Conclusion

The effects of backward velocity will be greater in magnitude considering the median across the entire Amazon biome. This striking pattern of backward velocity occurs both for climatic conditions with and without future analogs, with emphasis on a large expanse of no-analogs across central Amazonia. This indicates that high temperatures and changes in precipitation will exceed the historical variability that occurs in the biome, which may have effects on ecosystem functioning and services of floodplain forests. Despite this, the network of Protected Areas will be less exposed to the impacts of the backward velocity than unprotected areas emphasizing the importance of these areas as a tool in conservation. In contrast, for forward velocity impacts, the PA network will be slightly more exposed than unprotected areas indicating that the current spatial arrangement of the PA network is still not the most suitable for minimizing the impacts of climate redistribution. Furthermore, at both velocities, the potential impacts will be greater on ILs than on PAs. However, risks to PAs should also be emphasized as the range of no-analogs is greater in this land category. Therefore, despite the important role of PAs in reducing land use changes, they are not immune to the effects of climate change, and new management strategies, specific to each area and that allow adaptation to global changes, will be necessary. Despite the methodological limitations inherent to analyzes based on climate change metrics [57], it is urgent to refine information about the effect of global changes on biota in the tropics, With the input provided here, we hope to further improve our abilities to identify the different needs for adaptive management of PAs in the face of climate change, providing decision-makers with more personalized data for the conservation of biodiversity in the Amazon.

## Supporting information

S1 Fig Referenced territorial limits. Location of the Amazon biogeographic boundary (based on RAISG) and protected areas (based on WDPA).
(TIF)

S2 Fig Maps of the difference between projected and current climate variables (climatic delta) and historical annual variability. Spatial distribution of climate deltas (2050–1990) of temperature and precipitation and spatial distribution of historical annual variability of temperature and precipitation.
(TIF)

S3 Fig Nine-quadrant bivariate plot with a joint characterization of forward and backward velocities, where the range of velocity values is divided into low, moderate, and high categories using three equal area quantiles. (TIF)

S1 Table General circulation models (GCM) used for projections of bioclimatic variables. 5 General circulation models (GCM) from CMIP6 used to assess the future distribution of climate conditions. A multi-model ensemble was generated based on all the GCMs.
(XLSX)
