# Supplementary figures and images for: The climatic risk of Amazonian protected areas is driven by climate velocity until 2050
Source: PLoS One. 2023 Jun 22;18(6):e0286457. doi: 10.1371/journal.pone.0286457 (PMC10286990; doi:10.1371/journal.pone.0286457)

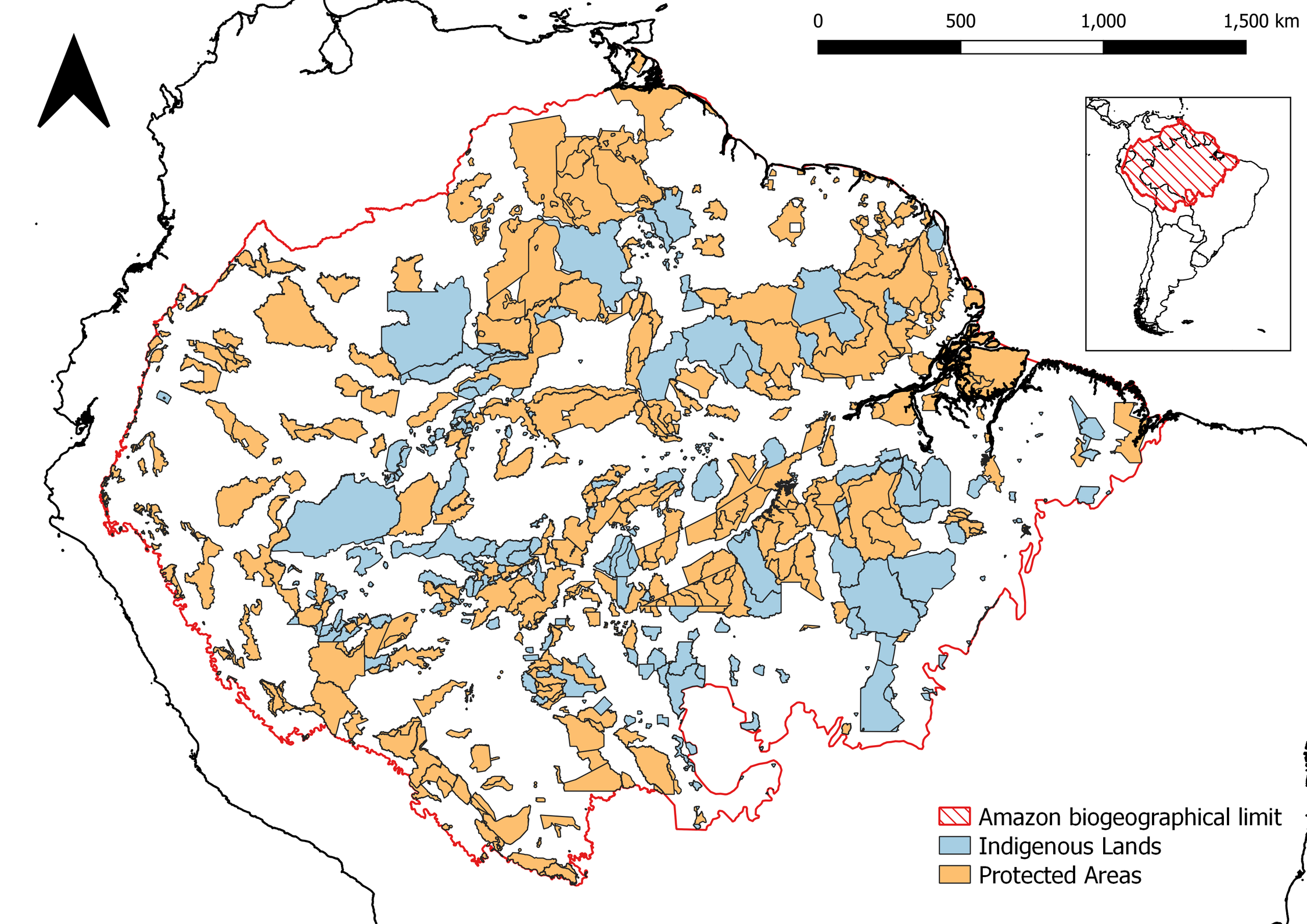

Supplement: S1 Fig — Location of the Amazon biogeographic boundary (based on RAISG) and protected areas (based on WDPA). (TIF) [file pone.0286457.s001.tif]

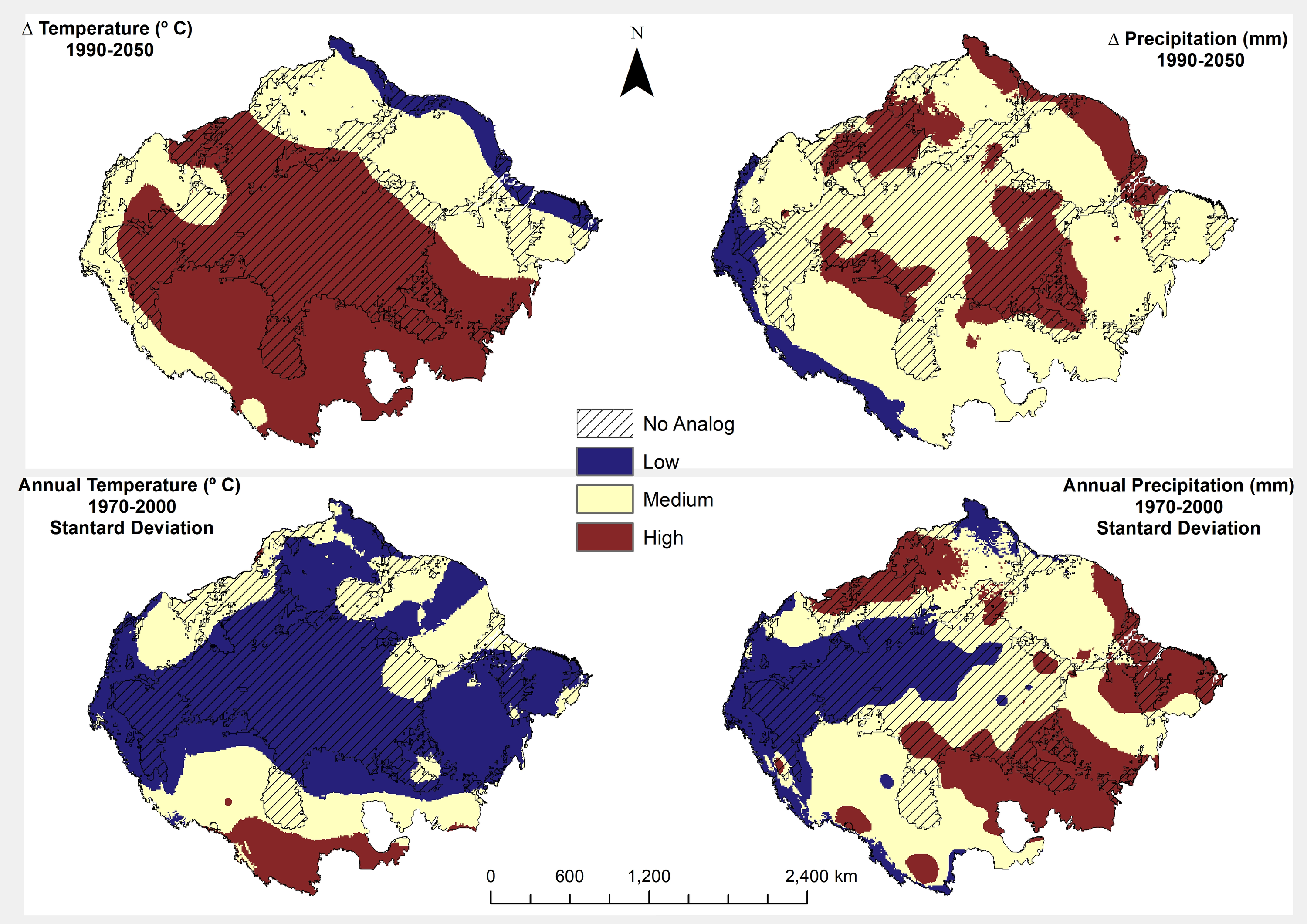

Supplement: S2 Fig — Spatial distribution of climate deltas (2050–1990) of temperature and precipitation and spatial distribution of historical annual variability of temperature and precipitation. (TIF) [file pone.0286457.s002.tif]

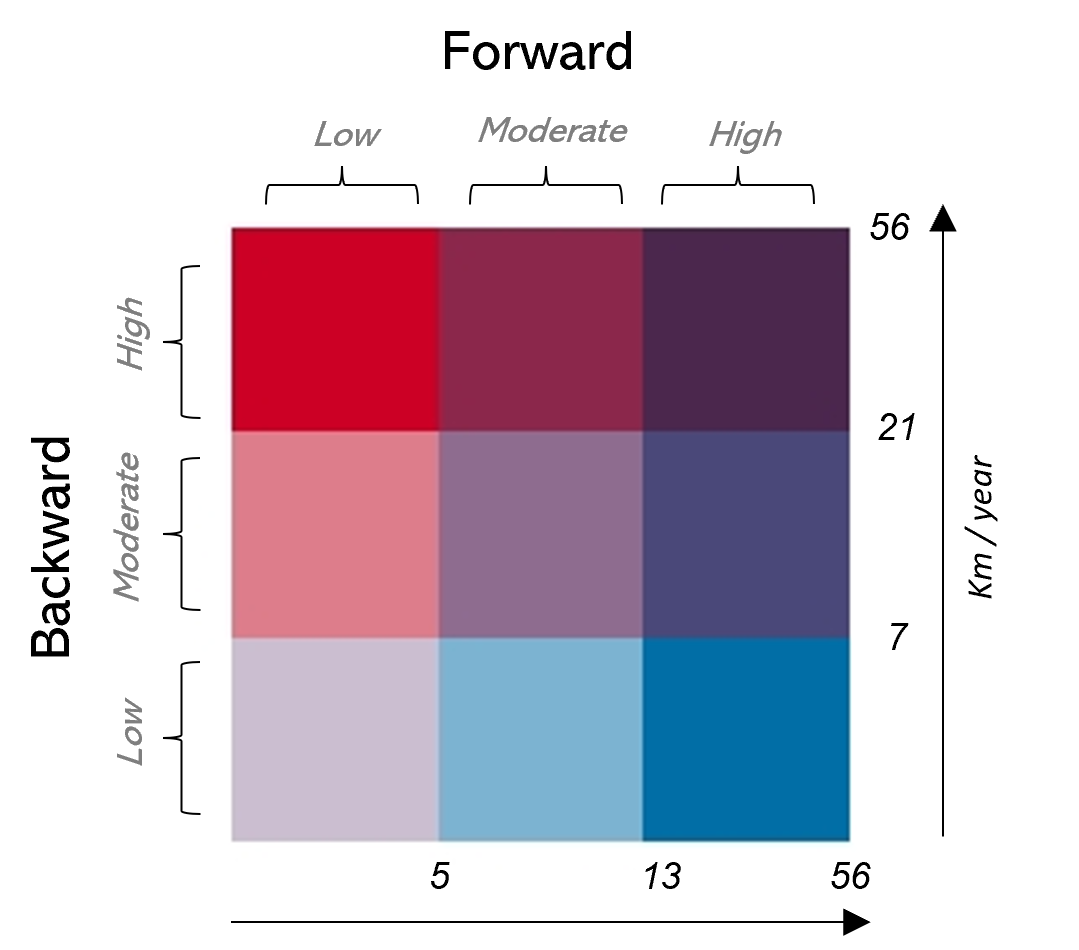

Supplement: S3 Fig — (TIF) [file pone.0286457.s003.tif]
